# Distance to the Neutral Face Predicts Arousal Ratings of Dynamic Facial Expressions in Individuals With and Without Autism Spectrum Disorder

**DOI:** 10.3389/fpsyg.2020.577494

**Published:** 2020-11-27

**Authors:** Jan N. Schneider, Timothy R. Brick, Isabel Dziobek

**Affiliations:** ^1^Institut für Informatik und Computational Science, Universität Potsdam, Potsdam, Germany; ^2^Berlin School of Mind and Brain, Humboldt-Universität zu Berlin, Berlin, Germany; ^3^Human Development and Family Studies and Institute for CyberScience, The Pennsylvania State University, State College, PA, United States

**Keywords:** arousal, face tracking, facial expression, autism, perception, perception differences, measure development

## Abstract

Arousal is one of the dimensions of core affect and frequently used to describe experienced or observed emotional states. While arousal ratings of facial expressions are collected in many studies it is not well understood how arousal is displayed in or interpreted from facial expressions. In the context of socioemotional disorders such as Autism Spectrum Disorder, this poses the question of a differential use of facial information for arousal perception. In this study, we demonstrate how automated face-tracking tools can be used to extract predictors of arousal judgments. We find moderate to strong correlations among all measures of static information on one hand and all measures of dynamic information on the other. Based on these results, we tested two measures, average distance to the neutral face and average facial movement speed, within and between neurotypical individuals (*N* = 401) and individuals with autism (*N* = 19). Distance to the neutral face was predictive of arousal in both groups. Lower mean arousal ratings were found for the autistic group, but no difference in correlation of the measures and arousal ratings could be found between groups. Results were replicated in an high autistic traits group. The findings suggest a qualitatively similar perception of arousal for individuals with and without autism. No correlations between valence ratings and any of the measures could be found, emphasizing the specificity of our tested measures. Distance and speed predictors share variability and thus speed should not be discarded as a predictor of arousal ratings.

## Introduction

Arousal is a frequently used and long-standing (Duffy, 1957) concept in physiology and psychology. For example, the core affect framework (Russell and Barrett, 1999; Russell, 2003) describes affective states only along the dimensions valence and arousal. In emotion research this framework is often used to quantify subjective affect experiences or observed affective states, for example from facial expressions (Britton et al., 2006). The valence axis of the core affect space shows systematic patterns with facial expression. For example, we found that valence ratings have a strong correlation (*r* = 0.87) with happiness ratings (Schneider et al., in preparation). The happiness of a facial expression in turn is mainly estimated from the mouth area in western cultures (Smith et al., 2005; Eisenbarth and Alpers, 2011; Jack et al., 2012), thus permitting an estimation of valence from facial expression via estimates of happiness.

The question as to how arousal can be characterized seems more complex. Emotional states such as surprise, anger or fear are consistently located in the high arousal range of core affect space (Russell, 1980; Reisenzein, 1994; Russell and Barrett, 1999; Fontaine et al., 2007; Hepach et al., 2011). These discrete emotions have been linked to expressive facial affect, for example in the works of Ekman (1992a,b). However, to the best of our knowledge no literature exists that examines the qualities of a facial expression which are used by an observer to directly determine levels of arousal. The current study proposes to fill this gap by identifying features of facial expressions consistently related to observer levels of arousal.

We first identified several likely candidate features; we here describe how these features can be computed efficiently from face tracking data and explore their intercorrelations. Based on the exploratory results, we selected two features for confirmatory testing, and examined them within and between neurotypical (NT) individuals and individuals diagnosed with Autism Spectrum Disorder (ASD).

### Arousal Cues From Facial Expressions

Observers can readily judge the arousal of a person displaying a facial expression, even in static images (e.g., Sato and Yoshikawa, 2007). The concept of arousal seems to be intuitively linked to physical activation. In fact, Russell and Barrett (1999) used the term activation in their seminal paper on core affect to describe this concept and state that other names of the concept have been “energy, tension [and] activity” in various theories of emotion. Two of these terms, energy and activity, seem also to be tightly related to the idea of movement and dynamics, and indeed the literature shows that dynamic stimuli produce increased arousal ratings by observers compared to static stimuli (Detenber et al., 1998; Sato and Yoshikawa, 2007; Sato et al., 2008). Thus motion information seems to provide important cues for arousal perception.

Several features of the dynamic, but also of the static aspects, of facial expressions could potentially be used as cues for arousal by an observer. A likely static feature might be the distance from the neutral face. For example, a happy expression is typically characterized by an upward pull of the corners of the lip and a rise of the cheeks instantiated predominantly by the zygomaticus major muscle (Wingenbach et al., 2020) of the face. The resulting shape changes of the lips and cheeks differ in the amount of displacement among different happy expressions, such as between small and large smiles. The distance of a facial expression to the neutral face is the total quantity of shape change across the face. Displacement in the context of facial expression can be considered a static feature, because it can be assessed from a still image, given that the observer has sufficient knowledge about human faces to approximate what the actor’s neutral face might look like.

Another potential cue for arousal could be the velocity of a facial expression. In the example of a happy expression, this is a measure of how fast the lips and cheeks move as the expression is made and relaxed. The velocity of an facial expression can be computed as the combined velocity of all parts of the face. Yet another arousal cue might be the acceleration of a facial expression, which corresponds to the change in velocity over time—a measure of how suddenly the expression appears or disappears. Velocity and acceleration are dynamic features, because they measure characteristics of movement—an observer would need a sequence of images or a video to estimate them. Mathematically, the velocity is the first derivative of displacement with respect to time, and acceleration its second derivative.

All of these measures can be calculated at every time point during a facial expression, e.g., for every frame of a video (with minimal loss of information for the dynamic features in a calculation with discrete time steps). An observer of a facial expression, however, has the ability to provide only a single arousal rating for the whole facial expression, which hints at an aggregation of facial expression information across time. This may be similar to gist representations in memory (Thompson, 2014), which capture essential information of complex phenomena and guide decision making. It is unclear, however, which qualities of a facial expression remain in its gist representation and how these are utilized to give accurate arousal ratings. The aggregation process for facial movement could take any of several forms. For example, an observer could be most sensitive to the average movement over the entire expression, or only keep track of the fastest or furthest extent that the expression reaches. In terms of aggregating measures of the frames of a video this would correspond to averaging measures across frames or taking the maximum across frames, respectively.

### Face Processing in ASD

Autism Spectrum Disorder is a developmental condition characterized in the DSM-5 by pervasive social dysfunction, stereotyped and repetitive behaviors and interests (American Psychiatric Association, 2013). Some of the disorder’s dysfunctions in communication and interaction behavior have been hypothesized to be the result of impairments in specific cognitive processes, for example in emotion recognition (Høyland et al., 2017). Hence, a considerable amount of research has focused on face perception and emotional facial expression recognition in autism (Harms et al., 2010; Lozier et al., 2014). Eye tracking studies have found less attention toward faces (Riby and Hancock, 2009; Kirchner et al., 2011) and increased attention toward bodies and objects in individuals with autism (Klin et al., 2002). Individuals with ASD looking at faces have been found to show patterns of avoidance of the eye region and to predominantly focus on the mouth area (Klin et al., 2002; Jones et al., 2008; Kliemann et al., 2010), although these findings have also been challenged in the recent literature (Guillon et al., 2014).

Autism Spectrum Disorder has been found to be accompanied by difficulties in emotion recognition, for example from facial expressions (Rump et al., 2009). Uljarevic and Hamilton (2013), found a mean effect size of *d* = 0.41 for the difficulty of emotion recognition in autism in a meta-analysis. Not much is known about the underlying mechanism of emotion understanding deficits. One possible explanation for the difficulties may lie in abnormalities in lower level sensory processing such as in motion perception.

### Perception of Movement in Autism Spectrum Disorder

Although differences in movement perception between NT and ASD individuals are frequently reported, results about the exact nature of those differences are often contradictory. Some studies found a reduced sensitivity for motion detection in autistic individuals (Milne et al., 2002; Bertone et al., 2003; Robertson et al., 2014), but others found an enhancement under certain conditions. For example, Foss-Feig et al. (2013) found superior motion perception in children with ASD compared to NT children for small and large stimuli in a high contrast condition, but not in a low contrast condition. Some authors reasoned that an impairment might only exist for biological motion, and indeed some (Blake et al., 2003; Nackaerts et al., 2012), but not all, studies showed an impairment of biological motion perception in autism (Saygin et al., 2010; Rutherford and Troje, 2012). Freitag et al. (2008) found a decreased activity in response to biological motion in temporal and parietal areas as well as in the anterior cingulate gyrus for ASD individuals. Additionally, they found an increase in reaction time for ASD individuals when viewing stimuli of biological motion. However, they attributed these differences to difficulties in higher-order motion perception or the integration of complex motion information in ASD, and not to the biological nature of the motion *per se*.

In light of the presented evidence for abnormal motion, face and emotion processing associated with ASD, it seems likely that perception of arousal from facial expressions is affected in individuals with ASD and hence may also differ between the NT and ASD population. These research hypotheses guided the following two studies.

## Study 1: Measure Selection

Recent advancements in computer vision software allow frame-by-frame tracking of the entire face, and therefore provide an efficient and automatic way to measure facial movements. In the following such face tracking data are used to determine which features of facial expressions are predictive of raters’ arousal judgments. First, we examined the correlation between various measures that could serve as arousal cues in an exploratory way and then tested two selected measures (average distance from the clip neutral face and average speed) on a separate data set with confirmatory analyses. The following describes the initial measures that seemed to be likely candidates for arousal cues. The section provides detailed information on the calculation of these candidate measures. It then describes the process used to select the two measures that were later tested in the confirmatory analyses in Study 2.

### Methods

#### Materials

##### Video data sets

A data set of 120 video clips showing facial expressions from 40 different emotional categories (Supplementary Table S1) produced by three female actors was used for measure selection. These video clips were part of a larger set of videos, which were recorded in an effort to produce a set of ecologically valid facial expression stimuli. The videos were recorded at the film studio of the Humboldt University, Berlin, Germany, in cooperation with its Computer and Media Service. The 40 emotion categories that the actors were to display were selected because they occur frequently in everyday life and are roughly evenly distributed across the valence and arousal dimensions (Hepach et al., 2011). These categories also included but were not limited to Ekman’s six basic emotions (Ekman, 1992a). The actors received specific emotion induction instructions that included situations in which the emotion to be portrayed occurs and information about physiological changes associated with the emotion. Video clips were validated by experts and showed high emotion recognition rates and good believability. Each video clip shows an actor’s face centered in front of a gray background (Supplementary Figure S1). The actor first displays a neutral facial expression, moves to display an emotional facial expression of the selected emotion and subsequently returns to a neutral expression. A complete description of the stimulus material production can be found in (Kliemann et al., 2013).

##### Face tracking data

Tracking data for all videos was acquired using the software OpenFace (Baltrusaitis, 2018). OpenFace provides the *x*- and *y*-coordinates of 68 landmarks that are placed on the face for each frame of a facial expression video. All expressions were then normalized to a common frame of reference using Generalized Procrustes Analysis to remove differences due to the location on the frame or the overall size of the face in the video.

#### Measures

The measures that we investigated in this study are based on a measure of displacement and its time-derivatives. This initial measure of displacement must be relative to a “home base,” here called the baseline face, from which the displacement is calculated. In our exploratory analysis, we examined two different baseline faces: the clip neutral face and the actor’s mean face, which are described in the following.

##### Clip neutral face

**FIGURE 1:**
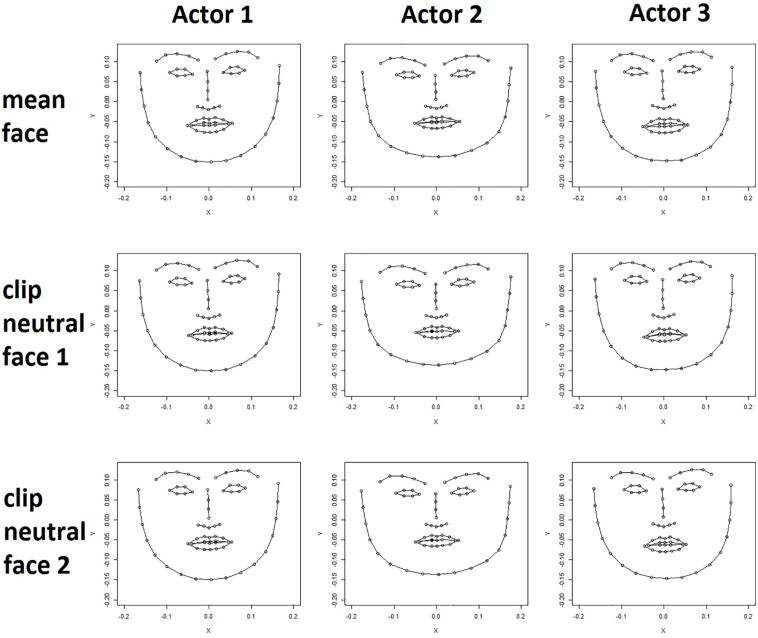
Actor mean face (upper row) and two clip neutral faces are shown for three actors (female, male, female). Clear differences in face shape can be seen between the actors. Differences between an actor’s mean face (upper row) and their clip neutral faces (bottom rows) are less apparent but nevertheless visible, for example, in the shape of the mouth.

For each clip the locations of points representing the neutral face of the actor featured in the clip were extracted from the first frame of the raw video clip. Even after the normalization procedure the neutral faces of an actor extracted from different video clips differed slightly in expression and angle toward the camera as is shown in Figure 1 (lower rows).

##### Actor mean face

The tracking data of all frames of an actor were averaged for each coordinate of each point to calculate a mean expression for each actor. Figure 1 (upper row) shows the mean actor face for three different actors. Differences in the shape of facial features, for example the mouth, and differences in the general expression are subtle but clearly visible. Importantly, the mean expression differs from the neutral expression in that the mouth is often slightly open, and the lips turned either upward or downward depending on the particular actor.

##### Understanding derivatives

One common means of understanding displacement and its derivatives is using the metaphor of a vehicle. Consider a person who rides a bicycle from point A to B. We can calculate their displacement by subtracting the location (in e.g., latitude and longitude) of point A from the location of point B. This displacement is a vector quantity (latitude, longitude) that has both a direction (e.g., north) and a length. The length of this displacement vector is called the (linear) distance between point A and B and a scalar quantity. Assuming the cyclist biked in a straight line from A to B without needing to turn, this distance would be the value shown on the mileage counter on their bike.

(1) $$\text{d}\,\left({\text{p}}_{f,}\,{\text{p}}_{f+a}\right)=\,\sqrt{{\left({\text{x}}_{p_{f+a}}-{\text{x}}_{p\,f}\right)}^{2}+{\left({\text{y}}_{p_{f+a}}-{\text{y}}_{p\,f}\right)}^{2}}$$

##### Distance measure: root mean squared deviation

Like the cyclist, the distance from a baseline can be computed for each face tracking point, now in terms of the distance along the *x* and *y* axes of the screen. Equation 1 shows this calculation, called the Euclidean Distance, for a single face tracking point *p* in two frames *f* and *f+a*. The result here is a single scalar value, representing the mileage counter for that point of the face.

If the cyclist was just one of 68 cyclists traveling the roads at a given time, we would need to accumulate the amount of movement covered by all of them. A simple mean is insufficient because they might travel in different directions and it would be undesirable to have a cyclist traveling south to “cancel out” the efforts of one traveling north. To capture the simultaneous deviation of all face tracking points of an facial expression from a baseline face (the clip neutral face or the actor mean face), we therefore computed the root mean square deviation (RMSD; Eq. 2). The RMSD of a facial expression is useful in this context, because it expresses the total distance in structural movements between two automatically tracked facial expressions, a good approximation of the total amount of movement required to change one expression into the other.

(2) $$\text{RMSD}\,\left(\text{f},\text{f}+\text{a}\right)=\,\sqrt{\frac{\sum_{\text{p}=0}^{\text{N}}\text{d}\,{\left({\text{p}}_{\text{f}},{\text{p}}_{\text{f}+\text{a}}\right)}^{2}}{\text{N}}}$$

##### Speed measure: root mean squared speed

If we knew how long it took the cyclist to get from point A to point B we could calculate their velocity simply by dividing their displacement by that time. Velocity is the change in displacement over time and has a directionality that indicates in which direction this change occurs. In our example, it occurs in the direction from A to B. The length of this velocity vector pointing from A toward B is called speed.

Similarly, we can compute the speed of each face tracking point. Equation 3 shows how the velocity $\vec{v_{p}}$ of a point p can be approximated over a time interval from frame *f* to Frame *f+a*. For subsequent frames *a* corresponds to 1. It is easy to see then, that the speed of a point between subsequent frames is equal to the Euclidean distance and can therefore also be calculated by Eq. 1.

(3) $$\vec{{\text{v}}_{p_{f,f+a}}}=\,\frac{{\text{p}}_{f+a}-{\text{p}}_{f}}{\left(\text{f}+\text{a}\right)-\text{f}}$$

To quantify the overall speed between two frames the RMSD (Eq. 2) can now be used again. The resulting value is the root mean squared speed of all tracking points between those two frames. It follows from the Euclidean Distance, that resulting speed values cannot be negative and hence cannot cancel out, if tracking points are moving in opposite directions.

##### Acceleration measure: root mean squared acceleration magnitude

Acceleration is the change in velocity over time. To calculate the acceleration for our cyclist we would need extra information, for example information about the location of some point S along the way of the cyclist and when they reached it. Then we could calculate the velocity of the cyclist between point A and S and between S and B. Then, we could use these two velocity vectors to calculate the acceleration of the cyclist between A and B by subtracting the first from the second and dividing the result by the time it took the cyclist to get from A to B.

Similarly, for each face tracking point p acceleration vectors between a frame *f* and the frame two frames after, *f+2*, were approximated by the velocities between the frames *f* and *f+1*, and *f+1* and *f+2*, as shown in Eq. 4. Equation 4 therefore describes the acceleration of a tracking point between a frame and the frame two frames after. To calculate the magnitude of the acceleration vector, the Euclidean distance (Eq. 1) of the respective velocity vectors can be used again. Root mean squared acceleration magnitude is therefore computed by computing RMSD(v_*f*_,v_*f+2*_) following Eq. 2. Once again, the squaring of acceleration magnitudes means that positive and negative acceleration accumulate rather than canceling each other out.

(4) $$\vec{{\text{a}}_{p_{f},p_{f+2}}}=\,\frac{\vec{{\text{v}}_{{\text{p}}_{f+1,},p_{f+2}}}-\,\vec{{\text{v}}_{p_{f},p_{f+1}}}}{\left(\text{f}+2\right)-\text{f}}$$

##### Aggregation of measures

The measures described above were calculated frame-wise or between subsequent frames in the case of the speed and acceleration magnitude measures. This results in a sequence of values for each video clip. It is possible that people remember the average distance, speed or magnitude of acceleration over the course of the whole clip, implying that the mean would be an appropriate aggregation. Alternatively, it may be that the peak of these is the most memorable part, and therefore has the strongest influence. Accordingly, we used the mean and maximum function to aggregate the frame-wise values into a single value.

#### Data Analysis

We first examined the correlation between the candidate measures of facial displacement and motion. The measures distance to the actor’s mean face, distance to the actor’s clip neutral face, speed and acceleration were calculated for all videos. These four types of measures were aggregated by the mean and maximum function each. This resulted in eight variables that were each observed 120 times, i.e., for each video clip. Based on that, we calculated the Pearson correlation matrix of these variables.

### Exploratory Results and Discussion

**FIGURE 2:**
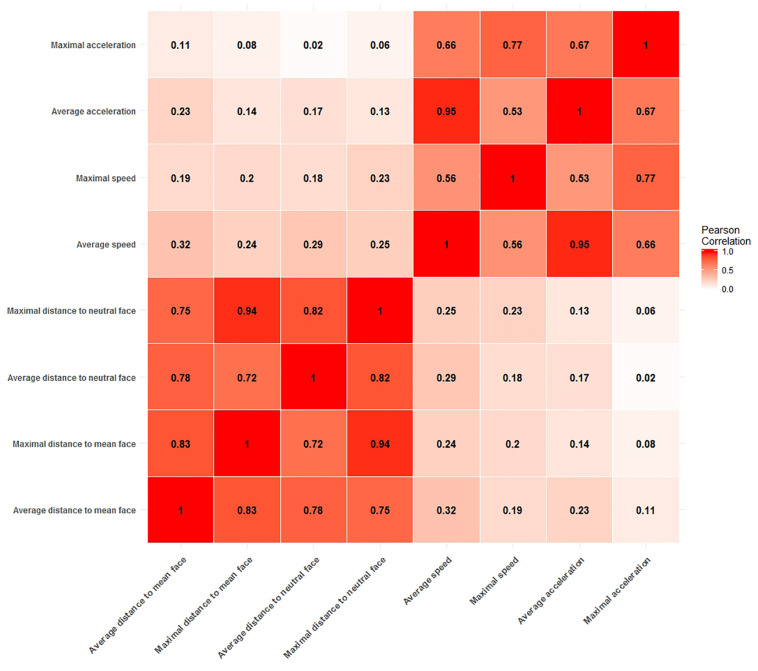
Correlation matrix of measures for potential arousal cues. Two blocks of moderate to strong correlations are clearly visible. One for all distance measures and the other for all speed and acceleration measures.

Figure 2 shows the Pearson correlations between potential arousal cue measures. From the correlation matrix it is evident that all distance measures are highly correlated with each other, with correlations ranging from *r* = 0.72 (average distance to neutral face and maximal distance to mean face) to as high as *r* = 0.94 (maximal distance to mean face and maximal distance to neutral face). Likewise, all measures of speed and acceleration are moderately to strongly correlated with values ranging from *r* = 0.53 (maximal speed to average acceleration) to *r* = 0.95 (average acceleration to average speed).

This clear divide between measures of static information, i.e., all distance measures and measures of dynamic information, i.e., measures of velocity and acceleration is of particular importance. High collinearity between predictors in a model is to be avoided, as it leads to unstable estimates of regression coefficients and complicates the interpretation of coefficients, since it is not possible to assign the explained variance in the dependent variable to one of the predictors. Therefore, our strategy was to select one measure from each block of correlations to use them in confirmatory analyses.

The correlation between the average acceleration measure and the average speed measure (*r* = 0.95) is especially noticeable, because such a strong correlation is not expected from the general relationship of speed and acceleration. In order to avoid problems with collinearity, we chose not to include any acceleration measure into our analyses. For the same reason we also only picked one variable for distance and speed each. We chose the average distance to the clip neutral face as the measure to quantify the distance to a ground face and the average speed to quantify the speed information. Given the strong correlations between measures, the choice of the specific measures does not carry too much weight as highly correlated measures carry the same information for the most part and would therefore behave similarly as predictors of arousal. However, we chose two measure which both used the mean function for aggregation of the time series information for the sake of comparability and because a mean aggregation is expected to be more robust to outliers in the time series information than the aggregation by the maximum function.

## Study 2: Predictors of Arousal

The presented exploratory investigations of Study 1 were conducted to pick measures that could act as cues for arousal prediction. We examined a number of measures and their intercorrelation structure, and selected a subset of predictors that we deemed to be representative, meaning they cover much of the explanatory power of other candidates, and distinct, meaning they do not overlap with the explanatory power of other chosen candidates. Our final feature set included only the average root mean square deviation to the clip neutral face as a measure of displacement and the average root mean square speed as a measure of speed. In Study 2, these measures were tested on their correlation with arousal ratings in NT and ASD individuals in confirmatory models.

We expected the distance to the neutral face and the speed of an facial expression to correlate with the arousal ratings from NT and ASD individuals. We furthermore expected the distance to the neutral face and the speed of an facial expression to correlate with the differences in arousal ratings between the groups. Additionally, we were interested in the arousal-specificity of the predictors. We deemed it possible that they could be general markers that provide information for a multitude of emotional judgments, for example also for the valence perception of the subjects. Therefore, we also tested for a correlation of the predictors with valence ratings within and between groups.

### Methods

#### Participants

Four hundred and one neurotypical (NT) participants (286 females, mean age 27.65 ± 7.74; 115 males, mean age 29.9 ± 9.83; sample mean age: 28.30 ± 8.44, age range 15–64) were recruited in an online study on emotion perception through online advertisements. NT participants were included if they were either female or male native German speakers. Nineteen participants (9 females, mean age 34.89 ± 13.54; 10 males, mean age 35.6 ± 7.35; sample mean age: 35.26 ± 10.42, age range 22–56) with a diagnosis of ASD were recruited through the collaborating autism outpatient clinic of Charité - Universitätsmedizin Berlin. All of the participants were diagnosed according to ICD-10 criteria for Asperger syndrome and Autism (World Health Organization, 1993). The diagnostic procedure included the Autism Diagnostic Observation Schedule (Lord et al., 2000) and the Autism Diagnostic Interview – Revised (Lord et al., 1994), if parental informants were available (*n* = 11).

An additional sample of 41 (11 male, 2 transgender) German speakers with high autistic traits (HAT) were collected from an autism online forum^1^. All 41 individuals self-reported that they had an autism diagnosis. They also scored above the cutoff of 32 in the Autism Spectrum Quotient (Baron-Cohen et al., 2001) (*M* = 39.58, *SD* = 3.84). Given, however, that we could neither verify their diagnosis nor assess the diagnostic protocols or instruments that were used, this participant group was only used in follow-up analyses to replicate our initial results.

All of the participants gave informed consent via an online form before their participation, and the study was approved by the ethics committee of the Charité – Universitätsmedizin Berlin.

#### Materials

##### Video data sets

A data set of 80 video clips also taken from the large video data set described under “Methods” section in Study 1 (and non-overlapping with the 120 videos from that study) was used. The video clips were cut to be 4 s long each, so that they contained the actor’s peak expression. 12 actors (six males, six females, age range: 21–64) which showed expressions from 21 emotion categories (Supplementary Table S1) appeared in this subset of the data. Videos were selected to capture emotion categories that equally spanned emotion space and difficulty space, based on emotion and difficulty ratings collected in a previous study (Schneider et al., in preparation). To achieve this, the mean ratings of 40 emotion categories were clustered with a hierarchical clustering approach using the R (Core Team R, 2017) function hclust. This resulted in 19 clusters. Then, up to two emotion categories per cluster were selected as representatives for the whole cluster.

##### Face tracking data

Tracking data for all facial expression videos was acquired and processed as described in the measure selection section above. Videos used in the present study consisted of 100 frames each, resulting in 6800 coordinates per video and consequently 13600 data points per video.

#### Procedure

Data for NT and ASD participants were collected in separate data collection efforts. Initial data for NT participants were collected as part of another study (Schneider et al., in preparation), which contained a wide range of videos. Eighty representative videos (see the section “Materials: Video Data Set”) were selected to be rated by ASD individuals. As a result, the number of videos rated by each individual in the NT group varied from 1 to 6 rated video clips, while each individual from the ASD group rated 14 video clips. Consequently, the 80 videos were rated on average by 11.24 participants from the NT group and 3.33 participants from the ASD group. All data collection was carried out in German on the soscisurvey.de platform (Leiner, 2014). Participants filled out a demographics questionnaire. Then each participant was presented with a sequence of videos randomly chosen from the pool of 80 videos. After each video, participants were asked to rate the valence (from “unpleasant” to “pleasant”) and arousal (from “very calm” to “very aroused/excited”). Participants rated valence and arousal on visual analog scales that encoded locations on the scale as integers between 1 and 101 and which were anchored by pictures of the respective Self-Assessment-Manikin (Bradley and Lang, 1994). After rating each video, participants were shown the next video.

#### Data Analyses

Distance to the neutral face and facial expression speed were computed as explained in the measurement selection section and averaged across video clips. Valence and arousal ratings were averaged per clip for the neurotypical and autistic group separately. Even though each participant only rated 14 video clips at most and as a result a considerable amount of missing data is present, this missingness is by definition *missing completely at random* because participants were assigned randomly to videos. Therefore, this missingness pattern is by definition not related to any variables included in the study, and the missingness pattern induces no bias in our results. The data were investigated with three linear regression models: one for each group (neurotypical individuals and individuals with ASD) and a separate model to specifically capture the differences between groups. Equation 5 shows the regression equation used for the separate models for each group. Here, arousal ratings from each group (NT or ASD) for a given video are predicted from the distance and speed measures computed for that video. Respective regression coefficients are estimated for each group as indicated by the group index *G*. Equation 6 shows the linear regression model that was used to investigate differences between the groups. The dependent variable is the difference in arousal ratings between the NT and ASD group. Again, distance and speed coefficients are estimated.

(5) $$A\,r\,o\,u\,s\,a\,l_{G}=\beta_{0_{G}}+\beta_{1_{G}}\times D\,i\,s\,t\,a\,n\,c\,e+\beta_{2_{G}}\times S\,p\,e\,e\,\text{d}+\epsilon$$

(6) $$A\,r\,o\,u\,s\,a\,l_{N\,T}-A\,r\,o\,u\,s\,a\,l_{A\,S\,D}=\beta_{0}+\beta_{1}\times D\,i\,s\,t\,a\,n\,c\,e+\beta_{2}\times S\,p\,e\,e\,d+\epsilon$$

### Confirmatory Results

#### Within-Group Results

**FIGURE 3:**
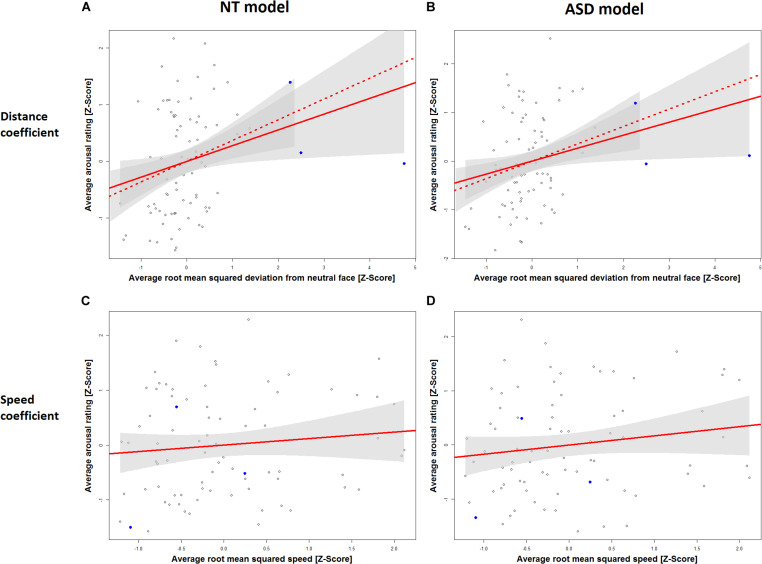
Added variable plots for the slope of the distance coefficient in the NT model **(A)**, the distance coefficient in the ASD model **(B)**, the speed coefficient in the NT model **(C)** and the speed coefficient in the ASD model **(D)**. Three outliers on the RMSD scale are marked in blue. Plots A and B show the regression slope for the distance coefficient including these outliers (solid line) and excluding them (dashed line). Ribbons (gray) show 95% confidence intervals.

Table 1 displays the results for the separate models for the NT and the ASD groups which were derived from Eq. 5. It shows a statistically significant effect of distance in the NT model (*p* = 0.0272) and the ASD model (*p* = 0.0324). The speed coefficient is not significant in both models (NT: *p* = 0.3393, ASD: *p* = 0.1709). The estimated standardized effect size for the distance coefficient is 0.28 in the NT model (Table 1) and 0.27 in the ASD model (Table 1). This corresponds to an average change in 5.93 and 5.79 points on the arousal scale, respectively, for each standard deviation of distance under a constant speed term (Table 1). The NT model explains 13% and the ASD model explains 15% of the variance in arousal ratings of the respective groups. The correlation between the distance measure and the speed measure (*r* = 0.51) was higher than we expected from the analyses in Study 1, which raised the possibility that predictive variance is shared between the two measures. Figure 3 shows the added-variable plots for the distance and speed predictors in the NT and ASD models. The plots for the distance predictor (Figures 3A,B) show three data points (blue) which are clearly separated from the main distribution and could thus be considered outliers. Calculating the mean without including them would put them at a distance of 4.9, 6.4, and 10 standard deviations from this new mean, which highlights the extremeness of these data points. These points also have high leverage scores (0.11, 0.17, 0.44; mean leverage: 0.038) resulting from their extreme values on the distance measure (*x*-axis) but not on the arousal measure (*y*-axis). Because they deviate from the trend apparent in the data they will have a strong influence on the slope of the regression coefficient for distance from the neutral face. The slopes of the estimated distance coefficients including (solid lines) and excluding (dotted lines) these outliers are shown for the NT and ASD model. One can see that without these three data points the slope would be more extreme, and hence the coefficients would be even larger than estimated by models on all data points. Specifically, without the outliers, the distance coefficient increases from 5.93 to 7.78 in the NT model (*p* = 0.00334) and from 5.79 to 7.73 in the ASD model (*p* = 0.00393). The explained variance of the NT model increases to 16% in the NT model and 18% in the ASD model. Predictors for speed remain non-significant in both models. Reasons for the extreme distance values are debated in the Discussion. There do not seem to be any outliers in regard to the speed measure.

**TABLE 1:** Regression models for the NT and ASD group predicting arousal ratings from distance to the neutral face and speed.

	*Dependent variable:*			
	
	Arousal rating NT	Arousal rating ASD	Arousal rating NT standardized	Arousal rating ASD standardized
	(1)	(2)	(3)	(4)
Distance [*Z*-score]	5.93*	5.79*	0.28*	0.27*
	[0.77, 11.09]	[0.58, 10.99]	[0.04, 0.52]	[0.03, 0.51]
Speed [*Z*-score]	2.53	3.67	0.12	0.17
	[−2.63, 7.69]	[−1.53, 8.88]	[−0.12, 0.36]	[−0.07, 0.41]
Intercept	47.60***	43.31***	−0.00	−0.00
	[43.19, 52.01]	[38.85, 47.76]	[−0.21, 0.21]	[−0.21, 0.21]
Observations	80	80	80	80
*R*^2^	0.13	0.15	0.13	0.15
Adjusted *R*^2^	0.10	0.12	0.10	0.12
Residual std. error (df = 77)	20.14	20.32	0.95	0.94
*F* Statistic (df = 2; 77)	5.53**	6.56**	5.53**	6.56**

Bold values highlight relevant significant effects. *p < 0.05; **p < 0.01; ***p < 0.001.

#### Between-Group Results

Table 2 shows the model of predicted mean arousal rating differences between the NT and ASD group as specified in Eq. 6. The only significant term in the model is the intercept of 4.29 (*p* = 0.00219). Because the dependent variable of this model is a difference score this indicates that there is a significant difference between the average arousal ratings of the NT and the ASD group: individuals with autism rated videos on average as showing 4 points (out of 101) lower in arousal than neurotypicals. Coefficients for distance and speed of the stimuli are not significant, however, indicating no group differences in the strength of correlation between the displacement and velocity displayed in the video and ratings of arousal.

**TABLE 2:** Regression models predicting differences in arousal ratings between the NT and ASD group from distance to the neutral face and speed.

	*Dependent variable:*	
	
	Arousal rating difference	Arousal rating difference standardized
	(1)	(2)
Distance [*Z*-score]	0.14	0.01
	[−2.97, 3.24]	[−0.25, 0.27]
Speed [*Z*-score]	−1.14	−0.09
	[−4.25, 1.96]	[−0.35, 0.16]
Intercept	**4.29^∗∗^**	−0.00
	[1.64, 6.95]	[−0.22, 0.22]
Observations	80	80
*R*^2^	0.01	0.01
Adjusted *R*^2^	−0.02	−0.02
Residual std. error (df = 77)	12.12	1.01
*F* Statistic (df = 2; 77)	0.31	0.31

Bold values highlight relevant significant effects. **p < 0.01.

#### Specificity of Predictors: Analyses of Valence

To gather evidence for the specificity of the tested predictors, we repeated our primary analyses with models as specified in Eqs (5) and (6), but with valence ratings instead of the arousal ratings for the dependent variable. In these two within-group models and one between-group model (Supplementary Tables S2, S3) none of the coefficients were significant.

### Exploratory Results

#### Individual Examination of Predictors

Because we suspected the two predictors to share variability in the dependent variable, we estimated coefficients for them in separate models. Table 3 shows models for the NT and ASD group which only contain a predictor for distance to the neutral face. Here, the distance coefficient increased to 7.21 and 7.66 respectively and was significant in the NT (*p* = 0.00208) as well as the ASD model (*p* = 0.00133). Both of these models explain 12% of the variance in the dependent variable. Table 4 shows models for the ASD and NT groups, which contain the speed measure as the only predictor. When not controlling for the effects of distance, the speed coefficients increased to 5.55 in the NT model and 6.62 in the ASD model, which were also significant (NT: *p* = 0.0194, ASD: *p* = 0.00596). Here, the NT model explains 7% of the variance in the dependent variable and the ASD model 9%.

**TABLE 3:** Regression models for the NT and ASD group predicting arousal ratings from distance to the neutral face only.

	*Dependent variable:*			
	
	Arousal rating NT	Arousal rating ASD	Arousal rating NT standardized	Arousal rating ASD standardized
	(1)	(2)	(3)	(4)
Distance [*Z*-score]	7.21**	7.66**	0.34**	0.35**
	[2.78, 11.65]	[3.15, 12.16]	[0.13, 0.55]	[0.15, 0.56]
Intercept	47.60***	43.31***	−0.00	0.00
	[43.19, 52.01]	[38.83, 47.79]	[−0.21, 0.21]	[−0.21, 0.21]
Observations	80	80	80	80
*R*^2^	0.12	0.12	0.12	0.12
Adjusted *R*^2^	0.10	0.11	0.10	0.11
Residual std. error (df = 78)	20.13	20.44	0.95	0.94
*F* Statistic (df = 1; 78)	10.15**	11.08**	10.15**	11.08**

Bold values highlight relevant significant effects. **p < 0.01; ***p < 0.001.

**TABLE 4:** Regression models for the NT and ASD group predicting arousal ratings from speed only.

	*Dependent variable:*			
	
	Arousal rating NT	Arousal rating ASD	Arousal rating NT standardized	Arousal rating ASD standardized
	(1)	(2)	(3)	(4)
Speed [*Z*-score]	5.55*	6.62**	0.26*	0.30**
	[0.99, 10.10]	[2.03, 11.21]	[0.05, 0.48]	[0.09, 0.52]
Intercept	47.60***	43.31***	0.00	0.00
	[43.07, 52.13]	[38.75, 47.87]	[−0.21, 0.21]	[−0.21, 0.21]
Observations	80	80	80	80
*R*^2^	0.07	0.09	0.07	0.09
Adjusted *R*^2^	0.06	0.08	0.06	0.08
Residual std. error (df = 78)	20.66	20.81	0.97	0.96
*F* Statistic (df = 1; 78)	5.70*	7.99**	5.70*	7.99**

Bold values highlight relevant significant effects. *p < 0.05; **p < 0.01; ***p < 0.001.

#### Replication of Results on a Sample of Individuals With High Autistic Traits

The sample size of the ASD group was small with *N* = 19 individuals. We repeated calculation of the within-group model with both predictors (Eq. 5) and the models with individual predictors on data from the HAT group, who self-reported to have been diagnosed with autism. In this group the distance coefficient is also significant (*b*_1_ = 6.15, *p* = 0.00916) in the model with both predictors and the coefficients for the distance and speed predictors are significant and increase when tested individually as previously seen in the ASD and NT group (Supplementary Table S4). We also calculated the difference model (Eq. 6) between the NT and the HAT group. We observe the same pattern of significance as found in the difference model between the NT and the ASD group, with only the intercept being significant (*b*_0_ = 4.64, *p* = 0.00047) and none of the predictors (Supplementary Table S5).

### Discussion

#### Predictors of Arousal

We found the average distance to the neutral face to be significantly predictive of the average arousal ratings in the NT and the ASD group, whereas speed showed no significance as a predictor in any of the confirmatory models. An additional exploratory analysis with a bigger sample (*N* = 41) of individuals with HAT who self-identified as autistic reproduced the significance pattern of the NT and ASD group with only the distance coefficient being significant.

Taken together, these results confirm distance to the neutral face as an important predictor for arousal in neurotypical individuals and individuals with autism. Arousal ratings can be and are frequently given to static facial expression stimuli. Our results might explain how this is possible even in the absence of movement information. The results suggest that observers, at least in part, rely on the displacement of the face, a feature that we captured with the distance to the neutral face measure.

Furthermore, the greater-than-expected correlation between the distance and speed measures (*r* = 0.51) highlights an issue of shared variability between the predictors, which complicates the simultaneous estimation of the effects of both predictors. This is exemplified by the explained variances of the models containing both predictors, which are lower than the sum of the explained variances of the models containing the predictors individually. In our data, distance to the neutral face is the stronger predictor as it explains more variance in the dependent variable than speed when tested on its own. Because the correlation between measures was much higher in Study 2 than in Study 1, and because our exploratory analyses indicated that either measure is significantly predictive of arousal, however, we are cautious in interpreting the specific breakdown of predictive power provided by the regression model. In this light and despite its non-significance in our confirmatory models, facial movement speed should not be discarded as a potentially important cue for arousal ratings of facial expressions.

To properly estimate the effects of both predictors independently, one could find or artificially create facial expression data where they are uncorrelated. On one hand, if the posed nature of our video clips is the primary reason for the correlation structure between speed and distance, then a data set of spontaneous expressions might be helpful in determining the unique contributions of distance and speed in emotion processing. However, Cohn and Schmidt (2004) found that the correlation between amplitude and duration was actually stronger in spontaneous smiles (*R*^2^ = 0.69) than in posed smiles (*R*^2^ = 0.09). If a similar relationship exists between distance (total change in amplitude) and speed (change in amplitude across duration), which this data suggests, efforts to research contributions of speed and distance to the neutral face independently in naturalistic data sets might actually be less effective, and researchers might be better advised to focus on artificially created stimuli. Importantly, however, Cohn and Schmidt’s (2004) work focuses expressly on happiness, and the results may not be the same for other emotions. For example, expressions of surprise (which are formed rapidly) may rely more heavily on dynamic information than do smiles. This also raises the question as to whether the specific emotion categories used in our study had an influence on our results. As discussed, detailed literature on the dynamic properties of emotion categories exists only for a few specific categories. However, the videos used in both presented studies were selected to cover a wide range of emotions that extends beyond Ekman’s basic emotions and which samples evenly from emotion space (see section “Materials” of Study 1). Therefore, we believe our results to be generalizable across emotion categories.

#### Differences in Arousal Perception Between Individuals With and Without Autism

We found the same pattern of significance in within-group models for the NT and ASD group. However, because this does not exclude the possibility of a significant difference in the size of the effects tested in the within-group models, we tested for group differences of distance and speed correlations with arousal. The only significant term in this model of difference scores between NT and ASD participants was the intercept. This means that participants with autism rated clips on average 4 points lower on the arousal scale, but it did not provide evidence for a difference in the strength of the distance and speed predictors between the groups. These results were replicated with the HAT sample, where the significance pattern stayed the same and the difference in arousal ratings as shown by the model intercept even increased slightly. These results indicate that individuals with autism make use of the same displacement and movement information as neurotypical individuals to judge arousal from facial expressions, and is consistent with the interpretation that arousal perception is qualitatively similar between groups. The current data are not sufficient to understand the precise nature of the change in intercept. On the one hand, it could be that people with autism in general show more cautious rating behavior. However, that no difference in valence ratings were found between groups constitutes evidence against this explanation. On the other hand, it is possible that while there is no qualitative difference in arousal perception, there may be a quantitative difference, such that individuals with autism are biased toward perceiving less arousal overall. This idea is consistent with prior work showing that autism is accompanied by aberrant empathy and theory of mind (Baron-Cohen and Wheelwright, 2004; Dziobek et al., 2008). If these empathy processes enhance the apparent arousal of an expression, we would expect a shift of this sort. Future work is required to determine whether such an enhancement exists and to what degree it explains lower arousal ratings in ASD. To this end, it could be tested whether arousal ratings of facial expressions are correlated with measures of empathy such as the Toronto Empathy Questionnaire (Spreng et al., 2009) or the Empathy Quotient (Baron-Cohen and Wheelwright, 2004) in individuals with ASD. To confirm the hypothesis a positive correlation between arousal ratings of facial expression video clips and an empathy measure should be established.

Individuals with ASD tend to avoid the eyes and focus instead more on the mouth when looking at faces (Klin et al., 2002; Jones et al., 2008). Similarly, evidence for a reduced integration of facial feature information has been found for individuals with autism for moving face stimuli, even when they attended to the eye region (Shah et al., 2016), which could also contribute to the lower arousal ratings of the ASD group. Assuming a model in which arousal information from different parts of the face is additive, either of these could explain lower arousal ratings in ASD, since in either case individuals with ASD do not attend to all the information present in the face. Both possible explanations and their respective contributions could be investigated in a study that correlates eye tracking data with arousal ratings. Another approach would be to systematically occlude parts of the faces that participants have to rate. Alternatively, measures similar to the ones used in our study could be calculated separately for upper and lower face regions.

#### Specificity of Arousal Predictors

Models that tested if the same predictors also predicted valence ratings within the NT and ASD groups or differences in valence ratings between the groups did not yield any significant coefficients. Also no mean difference in valence ratings between the groups was found. This suggests that the chosen predictors indeed have some specificity for arousal perception instead of capturing some general feature of facial affect.

#### Outlier Videos

Three video clips were identified as outliers in terms of the relationship between the distance measure and arousal. Two of the corresponding three video clips show the same actor, which could suggest that some idiosyncratic facial expression patterns of this actor are responsible for the extreme values on the distance to the neutral face measure for these videos. In fact, the majority of both clips show the actor’s head turned and eyes closed. Even though all tracking data were normalized in relation to a common coordinate system (Study 1; Methods: Face tracking data) artifacts from out-of-plane rotations of the head may still bias the computation of distance to the neutral face due to the focal length of the camera and the projection of the face onto the 2D plane captured by it. Similarly, our model of facial expression has six points per eye, actively upweighting the eyes’ influence. While this upweighting is effective at capturing the importance of the eyes in the understanding of facial expression, it may also provide some inconsistency here. Specifically, closed eyes may increase the distance of a clip from the neutral expression while *reducing* the apparent arousal by implying sleepiness or lethargy. While we do not wish to draw inferences from two outliers, future work should examine the specific effects of head movements, and the locations of movements that can be related to arousal in different situations. For example, eye-closing may carry different meaning than eye-opening for arousal. Similarly, it is plausible that head movements amplify the apparent arousal of an expression only when they are congruent with the expression. For example, turning ones gaze aside enhances the intensity of fear expressions, but reduces the intensity of anger expressions (Adams and Kleck, 2005). A similar relationship of perceived intensity and the interaction of head movement and emotional expression is conceivable. Finding an explanation for the outlier video closest to the main point cloud is not as straight forward. It shows the emotion category *enthusiasm* and differences from the three other videos displaying the same emotion in the data set are not immediately obvious. However, given its high average RMSD values it would be predicted to have higher arousal ratings. The expression of the actor seems less natural, i.e., more staged, compared to the other videos of the same emotional category, which might have led raters to assume that much of the activation displayed was a result of emotional masking (Brick et al., in preparation).

#### Design Choice and Future Work

Our selected stimuli showed facial expressions without any distractors—the background is solid gray and each video contains only the actor. Yet distractors frequently appear in situations when the interpretation of facial expressions is crucial, for example in social interactions, and might alter their perception. Distractors may be especially problematic for individuals with autism, who may have difficulty separating out distracting stimuli (Christ et al., 2011; Adams and Jarrold, 2012) and focusing their attention toward facial expressions (Klin et al., 2002; Riby and Hancock, 2009). Future work including distractors might find stronger differences between ASD and NT groups.

Although our results provide insight into the predictors of arousal, it has to be noted that all presented models only explain a small part of the variance of the arousal ratings. This poses the question how the unexplained part of the variance can be accounted for. It is likely that some part will be due to intersubjective differences in arousal perception and due to noisy measurements. However, features of facial expressions predictive of arousal other than the ones presented in this study are conceivable. For example, autonomic blood responses, as seen in skin tone might be such a feature as it is frequently paired with high-arousal states in everyday language (for example: “red with anger,” “to pale with fear,” “to blush with shame”). The recognition of certain emotional categories might themselves lead to an inference on arousal. Finally, other features from the tracking data, such as differences in face size and rotation that were removed by the normalization procedure we applied may influence arousal perception on their own. Future studies should investigate these variables separately and with appropriate stimuli.

### Limitations

Our study has some limitations due to its design. Although our participants were randomly assigned to videos, the design does not allow isolation of possible causes. As a result, no definite statement of causality can be made. Our sample also shows an average age difference (with the ASD group 6.97 years older on average) and a differential sex breakdown, with 29% male in the NT group and 53% in ASD. Both sex and age have known associations with general emotion perception (Ruffman et al., 2008; Isaacowitz and Stanley, 2011; Thompson and Voyer, 2014), and it is possible they have some effect on the group differences. However, conceptually it is not clear if and how these effects would be related to the task of arousal rating. At the individual rating level, however, the correlation of arousal rating with age is *r* = −0.004 for the NT sample and *r* = −0.02 for the ASD sample, and the correlation with sex is *r* = −0.006 and *r* = −0.10, respectively. Given such low correlations, we suspect that any bias induced by these demographic differences would be quite small.

## Conclusion

Arousal is a concept frequently used in emotion research and described to be related to physical activation and alertness (Russell and Barrett, 1999). In the core affect framework (Russell and Barrett, 1999; Russell, 2003) arousal is used as a dimension to describe emotional states and, among other applications, used to rate emotional facial expressions. However, the perception of arousal from facial expressions seems to be understudied and it is unknown which features of facial expressions have an influence on the arousal perception. In this study, we described characteristics of facial expressions, such as the distance to the neutral face, speed and acceleration magnitude, that are likely to be used for arousal estimation by the observer. We described in detail how these can be quantified with measures computed from face tracking data and discussed different methods of aggregating these measures over the course of a facial expression.

In Study 1, we first investigated the correlations among a set of potential measures. All measures for distance from a baseline face, and measures for speed and acceleration magnitude, respectively, showed moderate to strong correlations. Based on these results, we selected the average distance from the actor’s clip neutral face and the average speed of the expression as our best predictors. These two features were then used in Study 2 to predict arousal ratings from neurotypical individuals and from individuals with autism, and additionally to predict the differences in arousal ratings between those groups. Distance and speed each have some power to predict arousal, although the effect of speed disappears when controlling for distance in the NT and ASD sample. We found a statistically significant difference in the intercept of arousal ratings between the groups, but found no influence of the selected measures on the difference in arousal ratings. We could reproduce these within and between-group findings with an exploratory group of participants with high autistic traits who self-identified as having ASD. The results suggest that arousal perception in individuals with ASD is qualitatively similar to neurotypical individuals, and that differences in ratings may merely be a matter of degree. The predictors distance and speed seem to be specific predictors for arousal to some extent, as they do not predict valence ratings.

## Data Availability Statement

The raw data supporting the conclusions of this article will be made available by the authors, without undue reservation.

## Ethics Statement

The studies involving human participants were reviewed and approved by Ethikkommission Ethikausschuss am Campus Charité - Mitte Charitéplatz 1 10117 Berlin. Written informed consent from the participants’ legal guardian/next of kin was not required to participate in this study in accordance with the national legislation and the institutional requirements. Written informed consent was obtained from the individual(s) for the publication of any potentially identifiable images or data included in this article.

## Author Contributions

JNS developed the research idea with the help of TRB and ID. JNS, TRB, and ID developed the research design. JNS collected and analyzed the data under the supervision of TRB. JNS drafted the manuscript which was commented, discussed and reviewed extensively by TRB and ID. All authors contributed to the article and approved the submitted version.

## Conflict of Interest

The authors declare that the research was conducted in the absence of any commercial or financial relationships that could be construed as a potential conflict of interest.

## Footnotes

1 https://aspies.de/selbsthilfeforum
