# Supplementary material for: Distance to the Neutral Face Predicts Arousal Ratings of Dynamic Facial Expressions in Individuals With and Without Autism Spectrum Disorder
Source: Front Psychol. 2020 Nov 27;11:577494. doi: 10.3389/fpsyg.2020.577494 (PMC7729191; doi:10.3389/fpsyg.2020.577494)
Supplement: Supplementary file 1 [file Data_Sheet_1.pdf]

frontiers

Supplementary Material

Supplementary Table 1: *Emotion categories of the video data set. The original German label (left column) and the English translation (right column) are shown for all 40 categories of the video data set. The third column indicates if this emotion category was also used in Study 2.*

Original category name	English translation	Included in Study 2
amüsiert	amused	
angeekelt	disgusted	x
angstvoll	fearful	x
ärgerlich	angry	x
beleidigt	offended	
betroffen	affected	
beunruhigt	troubled	
dankbar	grateful	
eifersüchtig	jealous	
enthusiastisch	enthusiastic	x
entschuldigend	apologetic	
entsetzt	aghast	x
enttäuscht	disappointed	x
erleichtert	relieved	
erwartungsvoll	expectant	
frustriert	frustrated	
gekränkt	aggrieved	
gelangweilt	bored	x
heiter	happy	x
interessiert	interested	x
melancholisch	melancholic	x

mitleidig	compassionate	x
neidisch	envious	x
neugierig	curious	x
schuldig	guilty	
schwärmerisch	lyrical	x
stolz	proud	
traurig	sad	x
überrascht	surprised	x
verachtend	contemptuous	x
vergebend	pardoning	x
verlegen	embarrassed	
verliebt	inlove	x
verwirrt	confused	
verzweifelt	desperate	x
wehmütig	wistful	
wütend	furious	
zufrieden	content	
zuversichtlich	confident	x
zweifelnd	doubtful	

Supplementary Table 2: *OLS models for the NT and ASD group predicting valence ratings from distance and speed*

	Dependent variable:			
	Valence rating NT	Valence rating ASD	Valence rating NT standardized	Valence rating ASD standardized
	(1)	(2)	(3)	(4)
Distance [Z-Score]	2.66	-0.19	0.09	-0.01
	[-4.61, 9.92]	[-6.51, 6.13]	[-0.16, 0.35]	[-0.27, 0.25]
Speed [Z-Score]	0.09	1.49	0.003	0.06
	[-7.18, 7.36]	[-4.84, 7.81]	[-0.26, 0.26]	[-0.20, 0.32]
Intercept	43.47^{***}	44.12^{***}	0.00	0.00
	[37.25, 49.68]	[38.71, 49.53]	[-0.22, 0.22]	[-0.22, 0.22]
Observations	80	80	80	80
R^2	0.01	0.003	0.01	0.003
Adjusted R^2	-0.02	-0.02	-0.02	-0.02
Residual Std. Error (df = 77)	28.37	24.68	1.01	1.01
F Statistic (df = 2; 77)	0.36	0.13	0.36	0.13
Note:				$^{*}p<0.05$; $^{**}p<0.01$; $^{***}p<0.001$

Supplementary Table 3: *OLS models predicting differences in valence ratings between the NT and ASD group from distance to the neutral face and speed*

	Dependent variable:	
	Arousal difference	Arousal difference
	(1)	(2)
Distance [Z-Score]	2.84	0.23
	[-0.35, 6.04]	[-0.03, 0.48]
Speed [Z-Score]	-1.40	-0.11
	[-4.59, 1.80]	[-0.37, 0.14]
Intercept	-0.65	-0.00
	[-3.38, 2.08]	[-0.22, 0.22]
Observations	80	80
R^2	0.04	0.04
Adjusted R^2	0.01	0.01
Residual Std. Error (df = 77)	12.46	0.99
F Statistic (df = 2; 77)	1.53	1.53
Note:		$^{*}p<0.05$; $^{**}p<0.01$; $^{***}p<0.001$

Supplementary Table 4: *OLS models with distance to the neutral face and speed predictors tested together and individually for the HAT group.*

	Dependent variable:					
	Arousal ratings				Arousal ratings standardized	
	(1)	(2)	(3)	(4)	(5)	(6)
Distance [Z-Score]	**6.15****	**8.24*****		**0.31****	**0.42*****	
	[1.64, 10.66]	[4.30, 12.17]		[0.08, 0.55]	[0.22, 0.62]	
Speed [Z-Score]	4.10		**7.23*****	0.21		**0.37*****
	[-0.41, 8.61]		[3.20, 11.26]	[-0.02, 0.44]		[0.16, 0.58]
Intercept	42.96***	42.96***	42.96***	-0.00	-0.00	-0.00
	[39.10, 46.82]	[39.05, 46.87]	[38.95, 46.96]	[-0.20, 0.20]	[-0.20, 0.20]	[-0.20, 0.20]
Observations	80	80	80	80	80	80
R^2	0.20	0.17	0.13	0.20	0.17	0.13
Adj. R^2	0.18	0.16	0.12	0.18	0.16	0.12
Residual Std. Error	17.66 (df = 77)	17.87 (df = 78)	18.33 (df = 78)	0.90 (df = 77)	0.92 (df = 78)	0.94 (df = 78)
F Statistic	9.77*** (df = 2; 77)	16.26*** (df = 1; 78)	11.64** (df = 1; 78)	9.77*** (df = 2; 77)	16.26*** (df = 1; 78)	11.64** (df = 1; 78)
Note:						*p<0.05; **p<0.01; ***p<0.001

Supplementary Table 5: *OLS models predicting differences in arousal ratings between the NT and HAT group from distance to the neutral face and speed*

	Dependent variable:	
	Arousal difference	Arousal difference standardized
	(1)	(2)
Distance [Z-Score]	-0.22	-0.02
	[-3.13, 2.69]	[-0.28, 0.24]
Speed [Z-Score]	-1.57	-0.14
	[-4.48, 1.34]	[-0.39, 0.12]
Intercept	**4.64*****	0.00
	[2.15, 7.13]	[-0.22, 0.22]
Observations	80	80
R^2	0.02	0.02
Adjusted R^2	-0.003	-0.003
Residual Std. Error (df = 77)	11.36	1.00
F Statistic (df = 2; 77)	0.88	0.88
Note:		*p<0.05; **p<0.01; ***p<0.001

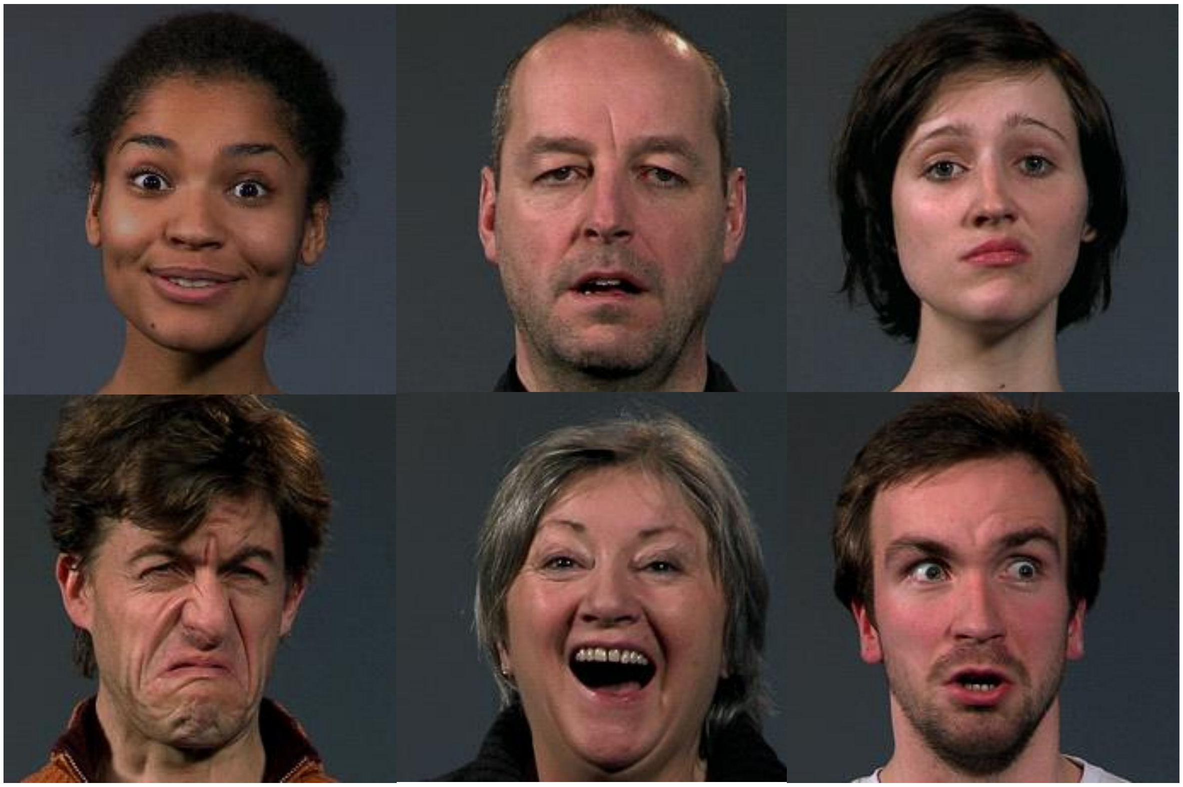

Supplementary Figure 1. Six exemplary still frames of the video data set from Study 2. The displayed expressions, from top left to bottom-right, are: curious, bored, contemptuous, disgusted, enthusiastic and fearful.
